# CDK2 regulates aminoglycoside-induced hair cell death through modulating c-Jun activity: Inhibiting CDK2 to preserve hearing

**DOI:** 10.3389/fnmol.2022.1013383

**Published:** 2022-10-13

**Authors:** Litao Tao, Neil Segil

**Affiliations:** ^1^Department of Stem Cell Biology and Regenerative Medicine, Keck School of Medicine, University of Southern California, Los Angeles, CA, United States; ^2^USC Caruso Department of Otolaryngology-Head and Neck Surgery, Keck School of Medicine, University of Southern California, Los Angeles, CA, United States

**Keywords:** aminoglycoside, gentamicin, ototoxicity, hair cell, apoptosis, CDK2, c-Jun

## Abstract

Sensory hair cell death caused by the ototoxic side effects of many clinically used drugs leads to permanent sensorineural hearing loss in patients. Aminoglycoside antibiotics are widely used and well-known for their ototoxicity, but the molecular mechanisms of aminoglycoside-induced hair cell death are not well understood. This creates challenges in our attempts to alleviate or prevent such adverse side effects. Here, we report a regulatory role of CDK2 in aminoglycoside-induced hair cell death. Utilizing organotypic cultures of cochleae from neonatal mice, we show that blocking CDK2 activity by either pharmaceutical inhibition or by *Cdk2* gene knockout protects hair cells against the ototoxicity of gentamicin—one of the most commonly used aminoglycoside antibiotics—by interfering with intrinsic programmed cell death processes. Specifically, we show that CDK2 inhibition delays the collapse of mitochondria and the activation of a caspase cascade. Furthermore, at the molecular level, inhibition of CDK2 activity influences proapoptotic JNK signaling by reducing the protein level of c-Jun and suppressing the gentamicin-induced upregulation of c-Jun target genes *Jun* and *Bim*. Our *in vivo* studies reveal that *Cdk2* gene knockout animals are significantly less sensitive to gentamicin ototoxicity compared to wild-type littermates. Altogether, our work ascertains the non-cell cycle role of CDK2 in regulating aminoglycoside-induced hair cell apoptosis and sheds lights on new potential strategies for hearing protection against ototoxicity.

## Introduction

Aminoglycoside antibiotics have an excellent bactericidal effect, especially against gram-negative bacteria (Forge and Schacht, 2000). However, it has long been known that aminoglycosides have ototoxic properties that induce the loss of sensory hair cells in the inner ear (Hinshaw et al., 1946). These specialized cells cannot be regenerated in the mammalian cochlea, hence resulting in permanent hearing loss (Groves, 2010). Our current understanding of the molecular mechanisms underlying aminoglycoside ototoxicity is very limited. Further investigation of these mechanisms is important not only to effectively alleviate the harmful side effects of these antibiotics but also to prevent hair cell death induced by other ototoxins, such as platinum-based antineoplastic drugs (Kros and Steyger, 2019).

The intrinsic mitochondria-caspase pathway of apoptosis has been found to be responsible for the loss of hair cells exposed to aminoglycoside ototoxicity (Cunningham et al., 2002, 2004). This route of programmed cell death requires gene transcription and *de novo* protein synthesis (Matsui et al., 2002). Thus, further elucidation of the upstream signaling pathways regulating intrinsic apoptotic gene and protein expression during aminoglycoside-induced hair cell death is essential to understand the mechanisms of aminoglycoside ototoxicity. Among such upstream pathways, the c-Jun N-terminal kinase (JNK) pathway has been implicated in aminoglycoside-induced hair cell death (Pirvola et al., 2000; Ylikoski et al., 2002). Upon aminoglycoside insult, JNK is activated by phosphorylation, and blockage of JNK activation protects hair cells from aminoglycoside-induced apoptosis (Pirvola et al., 2000; Ylikoski et al., 2002). After JNK activation, transcription factor c-Jun is phosphorylated by JNK and becomes transcriptionally active for the expression of downstream target genes, including *Jun* gene itself and the proapoptotic factor *Bim* (*Bcl2l11*) gene, to regulate cell death and survival (Angel et al., 1988; Biswas et al., 2007). It is unclear what other pathways interplay with JNK signaling during aminoglycoside-induced hair cell death.

Previously, we profiled transcriptome changes in hair cells in response to the aminoglycoside antibiotic gentamicin, finding thousands of genes significantly up- or downregulated 3 h after gentamicin treatment (Tao and Segil, 2015). As expected, the JNK pathway was identified as transcriptionally activated. We also noted an upregulation of cell cycle-related genes, including *Ccnb2* (*Cyclin B2*) and *Ccne2* (*Cyclin E2*) (Tao and Segil, 2015). Sensory hair cells are broadly known to be terminally differentiated, their postmitotic state actively maintained by high expression of cyclin-dependent kinase inhibitors (CKIs) and suppressed expression of cyclins (Chen et al., 2003; Laine et al., 2007). Our observation of cyclin genes being upregulated in these otherwise non-dividing cells is intriguing and may explain aminoglycoside-induced hair cell death by one or more evidence-based theories. One possibility is that the consequential activation of cyclin-dependent kinases (CDKs) by upregulated cyclins leads to aberrant cell cycle re-entry and ultimately p53-dependent apoptosis in aminoglycoside-treated cochlear hair cells, as observed in hair cells of CKI gene knockout animals (*p21^Cip^*^1^−/− and *p19^Ink^*^4^*^d^*−/−) (Chen et al., 2003; Laine et al., 2007). Another possibility is that cyclin gene upregulation and resulting CDK activation directly modulate the apoptosis of these hair cells in a non-cell cycle-related manner, as has been seen in neurons and other postmitotic cells (Konishi et al., 2002; Yuan et al., 2008; Vanden Bush and Bishop, 2011). Thus, we speculated that our observed upregulation of cyclins implies heightened CDK activity, and that CDK activity is involved in aminoglycoside-induced hair cell death in some way. Indeed, several independent drug screening studies have identified CDK inhibitors as potential otoprotectants (Ryals et al., 2017; Teitz et al., 2018; Menendez et al., unpublished data). However, how CDK activity regulates aminoglycoside-induced hair cell death was not clear.

In this study, we investigated the role of CDK activity in aminoglycoside-induced hair cell death, with a particular focus on the pathways governing the intrinsic mitochondria-caspase pathway of apoptosis. Using organotypic cultures of neonatal mouse cochleae, we show that when CDK2 activity is disrupted by either pharmaceutical inhibitors or gene knockout, more hair cells survive gentamicin treatment. Further investigation revealed that CDK2 inhibition delays both mitochondrial collapse and caspase cascade activation in hair cells after gentamicin treatment. Finally, we demonstrate that CDK2 inhibition leads to the reduction of c-Jun at the protein level and the suppression of gentamicin-induced transcriptional activation of c-Jun target genes, including *Jun* itself and the proapoptotic factor *Bim*. Collectively, our results suggest that CDK2 regulates aminoglycoside-induced apoptosis of postmitotic sensory hair cells through modulating c-Jun activity, and reveal an otoprotective effect of CDK2 inhibition against aminoglycoside antibiotics. Importantly, our hypothesis is supported by another line of evidence from *Cdk2*−/− animals. Compared to wild-type littermates, cochleae from *Cdk2*−/− mice were less sensitive to gentamicin treatment under both *ex vivo* and *in vivo* conditions.

## Materials and methods

### Animal strains and experimental usage of animals

*Atoh1-GFP* transgenic mice were obtained from Dr. Jane Johnson (Lumpkin et al., 2003). Mice carrying the *Cdk2* knockout allele were a courtesy gift from Dr. Philipp Kaldis (Berthet et al., 2003). Animal breeding and experimental usage were approved by the IACUC of House Research Institute (dissolved), Los Angeles, USA and the IACUC of University of Southern California, Los Angeles, USA. Both female and male animals were used in this study.

### Organotypic culture and drug treatment

Inner ears from postnatal day 1 (P1) mice were isolated under sterile conditions and further dissected in ice-cold Ca^2+^- and Mg^2+^-free PBS. The spiral ganglia, lateral wall and surrounding tissue were carefully removed under a dissection microscope, and the cochleae were cultured as previously described (Tao and Segil, 2015). Briefly, organs were mounted on a polycarbonate membrane (13 mm diameter, 1.0 μm pore size, SPI Supplies, West Chester, PA, USA) floating on DMEM/F12 medium (Invitrogen, Waltham, MA, USA) supplemented with 1% N2 (Gibco, Waltham, MA, USA) and 100 U/ml penicillin (Sigma-Aldrich, Burlington, MA, USA). Organ cultures were maintained under low oxygen conditions (37°C, 5% CO_2_ and 5% O_2_). Twenty-four hours after culture initiation, organs were treated with ∼0.5 mM gentamicin (Sigma-Aldrich, Burlington, MA, USA, ≥635 mg gentamicin base per 1,000 mg; 36 mg/ml for ∼50 mM stocking solution) for 3 h followed by fresh medium replacement.

Cyclin-dependent kinase inhibitors (Table 1) were added into the culture medium at desired concentrations together with gentamicin for 3 h, followed by washout and replacement with fresh medium containing only CDK inhibitors. SP600125 (Sigma-Aldrich, Burlington, MA, USA) was added in the culture medium at a concentration of 60 μM together with gentamicin for 3 h and kept in the medium after gentamicin washout. As controls for pharmaceutical inhibitors, an equivalent amount of DMSO (VWR, Radnor, PA, USA) was supplemented in the medium of control organ cultures. Texas-Red conjugated gentamicin (GTTR) mixture was prepared as described in literature (Steyger et al., 2003). To observe the accumulation of GTTR in hair cells, the GTTR mixture was diluted at 1:500 with the culture medium and then incubated with cochlear cultures for 30 mins before fixation and microscopy.

**TABLE 1:** Pharmaceutical cyclin-dependent kinase (CDK) inhibitors and their specificity.

Name	Specificity	IC50 (μM)					Conc. in assay (μM)^a^	Vendor
		CDK1	CDK2	CDK4	CDK5	CDK6		
Olomoucine	CDK1/2/5	7	7	>1,000	3	>250	600^b^	Alexis
PD0332991	CDK4/6	>10	>10	0.009	>10	0.015	20	Sigma
CVT-313	CDK2	4.2	0.5	215	n/a	n/a	320^c^	Calbiochem
RO3306	CDK1	0.035	0.34	2	n/a	n/a	50	Sigma
Roscovitine	CDK5	0.65	0.7	>100	0.16	>100	200	Sigma

^a^Highest concentration for each drug.

^b^Olomoucine showed protective effect at a concentration as low as 200 μM.

^c^CVT-313 showed protective effect at a concentration as low as 80 μM.

### Fluorescent immunostaining and cryosection

Cultured organs were fixed with 4% paraformaldehyde at room temperature for 30 mins, permeabilized with 0.2% Triton X-100 in PBS at room temperature for 2 h, and blocked with 10% donkey serum (Life Technology, Waltham, MA, USA) in PBS for 2 h. Rabbit anti-MYO6 antibody (Proteus Biosciences, Ramona, CA, USA), mouse anti-Parvalbumin antibody (Sigma), and mouse anti-p27 antibody (Neomarkers, Fremont, CA, USA) were diluted at 1:500 and incubated with organs at 4°C overnight. Alexa 594 or Alexa 488 conjugated secondary antibodies (Molecular Probes, Eugene, OR, USA) were diluted at 1:500 and incubated with organs at room temperature for 3 h. Samples underwent three 10-min washes after both primary and secondary antibody incubation.

For hair cell staining in mature cochleae, inner ears were isolated and surrounding tissues were removed to expose the round window and oval window. An opening was made at the apex of each organ with forceps. Next, inner ears were fixed with excessive 4% paraformaldehyde (>2 ml per organ) and rotated at room temperature overnight, followed by decalcification with 100 mM EDTA 2% paraformaldehyde (>10 ml per organ) for 24 h. Then, the whole tissue was stained as described above. Finally, cochleae were dissected out carefully and mounted on slides.

To prepare cross sections of cochlear organs, fixed samples were immersed in 30% sucrose (Sigma-Aldrich, Burlington, MA, USA) for 1 h, dipped into OCT (Sakura Finetek USA, Torrance, CA, USA) in a small mold, flash frozen with liquid nitrogen, and sectioned with a Leica CM3050S cryosection station at 10 μm thickness. Sections were stained with the same protocol described above.

### Auditory brainstem response and trans-tympanic membrane injection

Three-week-old mice were anaesthetized with an intraperitoneal injection of ketamine hydrochloride (50 mg/kg, Sigma-Aldrich, Burlington, MA, USA) and xylazine hydrochloride (10 mg/kg, Sigma-Aldrich, Burlington, MA, USA) and maintained at 37°C on a hot pad during the procedure. ABR recording was performed on both ears as described previously (Chen et al., 2003). Under anesthetic conditions, 25 μL 50 mg/mL gentamicin solution was injected through the tympanic membrane using a 30G needle into the right ear and the mice were kept lying on their left side until awake. Two weeks after gentamicin injection, recovery of the ear drum was confirmed under microscope after administering anesthesia, and ABR was performed on both injected ears and the contralateral control ears.

### Hair cell purification and quantitative-pCR

To obtain single cell suspension from neonatal cochleae, organs were digested with 0.05% trypsin (Invitrogen, Waltham, MA, USA) and 1 mg/ml collagenase (Worthington, Secaucus, NJ, USA) at 37°C for 8 mins and then triturated with a P200 pipette 300 times. The cell suspension was filtered with a 40 μm cell strainer (BD Biosciences, Franklin Lakes, NJ, USA) prior to FACS sorting with a BD FACSAria II machine. More than 95% of sorted GFP-positive cells were stained positive for MOY6 (data not shown). RNA was extracted from purified cells using Zymo Quick-RNA Microprep kit (Zymo Research, Irvine, CA, USA) and cDNA library was made by qScript reverse transcriptase SuperMix (Quanta Biosciences, Beverly Hills, CA, USA). Primers used to interrogate gene expression are listed as follows: *Rpl19* forward 5′-GGTCTGGTTGGATCCCAATG-3′, reverse 5′-CCCGGGAATGGACAGTCA-3′; *Jun* forward 5′-CCTTCT ACGACGATGCCCTC 3′, reverse 5′-GGTTCAAGGTCATG CTCTGTTT-3′; *Bim* forward 5′- CCCGGAGATACGGATTGC AC-3′, reverse 5′-GCCTCGCGGTAATCATTTGC-3′; *Gapdh* forward 5′-TGTGTCCGTCGTGGATCTGA-3′, reverse 5′-CC TGCTTCACCACCTTCTTGA-3′; *Bax* forward 5′-TGAAGA CAGGGGCCTTTTTG-3′, reverse 5′-AATTCGCCGGAGAC ACTCG-3′.

### Experimental designs and statistics

Organotypic cultures of cochleae were untreated (control) or treated with gentamicin. To quantify hair cell loss induced by gentamicin treatment, images were taken under a fluorescent dissecting microscope with 40× magnification and whole organ pictures were assembled from images of individual segments using Photoshop. The whole length of the cochlea was divided into four pieces with equal length: base, middle base, middle apex, and apex (∼300 μm from extreme base was excluded as the hook region). GFP-positive outer hair cells in each piece were counted to calculate the density along the length.

To test the protective effect of CDK inhibitors, explants were treated with gentamicin in the presence or absence of CDK inhibitors. GFP-positive or Parvalbumin positive outer hair cells were quantified at 24 h post treatment, and numbers of outer hair cells from gentamicin + inhibitor-treated organs were compared to those from organs treated with gentamicin and DMSO.

FITC-VAD-FMK (Promega, Madison, WI, USA) was used to monitor caspase cascade activation in live organ cultures from wild-type animals. FITC-VAD-FMK was added at 1:1,000 into the medium at 6 h post initiation of gentamicin or gentamicin + CVT-313 treatment. Fluorescent images were taken at 8-, 12-, and 24-h timepoints. FITC-positive hair cells in the base segments were counted, and organs treated with gentamicin and CVT-313 were compared to organs treated with gentamicin and DMSO.

Culture of organs from wild-type animals was used to monitor mitochondrial membrane integrity. Pre-warmed Rhodamine-123 (Sigma-Aldrich, Burlington, MA, USA, 1 mM working concentration) was added into the culture medium at 5.5-h timepoint, incubated for 30 mins, and images were taken from live cultures with a fluorescent dissecting microscope.

For quantitative-PCR, three technical replicates were made for each biological replicate and at least three biological replicates were collected. Gene expression fold changes were calculated by the delta-delta-Ct method using *Rpl19* as the internal control.

Student t-test was used for statistical analysis of the protective effect of Olomoucine. For statistical analysis of the dose-dependent protective effect of CVT-313 against gentamicin-induced hair cell death, one-way ANOVA test was first used to determine the existence of significant difference among dose groups and *post hoc* test (Bonferroni correction for adjusted-*p* value) was then used for statistical significance of individual pair-wise comparison. For statistical analyses in the rest of the study, we first used two-way ANOVA to determine the significances of main factors; if there was at least one significant main factor, we then performed simple comparison(s) within the significant factor either with *post hoc* test (Bonferroni correction for adjusted-*p* value) for multi-level factors or without *post hoc* test for two-level factors.

Error bars in figures indicate standard deviation, and OHC quantification data are expressed as Mean ± STD.

## Results

### Gentamicin induces cochlear hair cell apoptosis in organotypic culture

**FIGURE 1:**
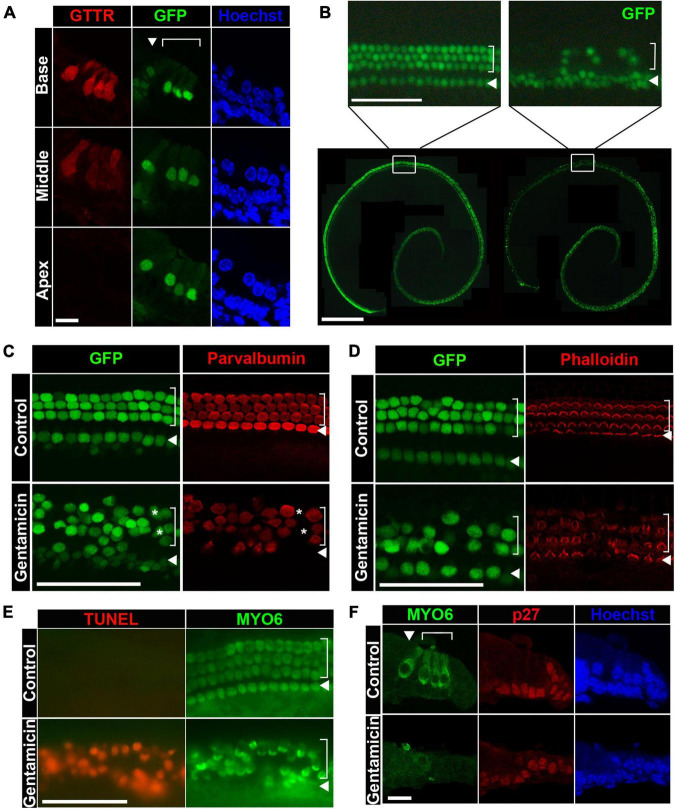
Gentamicin induces hair cell death through apoptosis in organ of Corti explant cultures. **(A)** Gentamicin accumulates specifically in hair cells with a base-to-apex gradient shown by cross-sections. GTTR, texas-conjugated gentamicin. GFP expression is under the control of *Atoh1* 3′ enhancer, labeling hair cells. **(B)** Gentamicin induces hair cell loss in organotypic cultures of cochleae in a base-to-apex wave shown by surface preps. *Bottom panel*, image of the untreated organ (*left*) and gentamicin-treated organs (*right*) at 24-h timepoint. *Top panel*, close-up image of untreated organ (*left*) and treated organs (*right*) at the middle-base position showing organized inner and outer hair cells in control organs and diminishing GFP-positive hair cells in gentamicin-treated organs. **(C)** Immunostaining of hair cell marker Parvalbumin showing that loss of GFP faithfully reflects the loss of hair cells. All hair cells are marked by GFP and stained positive for Parvalbumin in untreated control organs; and in gentamicin treated organs, Parvalbumin staining correlates with GFP staining. A few GFP-positive hair cells are Parvalbumin negative (asterisks), indicating that loss of Parvalbumin precedes the loss of GFP. Images were taken from the base region of the cochleae 18 h after treatment. **(D)** Immunostaining of hair cell stereocilia by Phalloidin shows the correlation between GFP loss and stereocilia disruption. Phalloidin labels the apical surface of hair cells, indicating healthy, undamaged stereocilia. Gentamicin-treated organs show similar patterns of GFP disappearance and stereocilia loss. Images were taken from the base regions of the cochlea at 18-h timepoint. **(E)** Hair cells die through apoptosis after gentamicin treatment. TUNEL (terminal deoxynucleotidyl transferase dUTP nick end labeling) signal is observed in hair cells (counterstained with MYO6) in gentamicin-treated organs at 24 h. Images were taken from the base regions of the cochlea. **(F)** Gentamicin specifically damages hair cells but not supporting cells. Hair cells are positive for MYO6 staining in cross-sections and supporting cells are labeled by p27 staining. Gentamicin-treated organs lose hair cells while supporting cells remain intact. Cultured cochleae were fixed at 24-h timepoint and sectioned for immunostaining, and representative images from the base region are shown. In both cross-sections and surface preps, inner hair cells are indicated by arrowheads and outer hair cells are indicated by brackets. Scale bars, 10 μm in panels **(A,F)**, 50 μm in top panel of panel **(B)** and in panels **(C–E)**, 500 μm in bottom panel of panel **(B)**.

To study the mechanisms of aminoglycoside ototoxicity, we used an established technique of organotypic culture of the mouse organ of Corti (Doetzlhofer et al., 2009; Rainey et al., 2016). We administered the commonly used aminoglycoside gentamicin to organ of Corti explants from Postnatal Day 1 (P1) *Atoh1-GFP* transgenic mice, in which sensory hair cells are labeled by GFP (Lumpkin et al., 2003; Figures 1A,B). Gentamicin has previously been shown to accumulate preferentially in sensory hair cells and not in supporting cells (Alharazneh et al., 2011). To test the selectivity of gentamicin absorption under our culture conditions, cultured organs were treated with Texas Red-conjugated gentamicin (GTTR) for 30 mins. Texas Red fluorescence was observed exclusively in outer and inner hair cells (verified by coincidence with Atoh1-GFP-positive cells) and showed a basal-to-apical (high-to-low) gradient (Figure 1A), as previously reported with submerged cultures of neonatal cochleae (Alharazneh et al., 2011). These results indicate that gentamicin accumulates specifically in hair cells in our organotypic culture system.

To measure the effect of gentamicin exposure on cochlear sensory hair cell survival, organ of Corti explants from *Atoh1-GFP* mice were cultured and treated with ∼0.5 mM gentamicin for 3 h, followed by culturing in gentamicin-free medium for an additional 21 h. Whole-mount preparations of gentamicin-treated explants displayed a basal-to-apical gradient of hair cell loss as indicated by the disappearance of GFP-positive hair cells, with outer hair cells (OHCs) more severely affected than inner hair cells (IHCs) (Figure 1B, right panel). Counts of GFP-positive hair cells confirmed the existence of a gradient of OHC destruction from base to apex [9.4 ± 1.6% (Mean ± STD) OHCs remaining in the base, 27.0 ± 2.3% in middle-base, 80.0 ± 5.9% in middle-apex, and 80.0 ± 7.8% in apex; *n* = 3; Supplementary Figure 1]. For quantification purposes, only OHCs were counted because of a low-level GFP mis-expression in inner phalangeal cells and border cells adjacent to IHCs. In contrast, untreated explants retained well-organized three rows of OHCs and one row of IHCs at the end of the 24-h incubation period (Figure 1B, left panel). To confirm that GFP disappearance is a faithful indicator of hair cell loss under our culture conditions, organ of Corti explants were stained with an antibody against the hair cell marker Parvalbumin (Heller et al., 2002) or labeled with Phalloidin to observe the stereocilia marking the apical surface of healthy hair cells (Li et al., 2008). We found that GFP-negative cells were also negative for Parvalbumin and Phalloidin staining (Figures 1C,D), corroborating our usage of GFP as a faithful indicator of hair cell loss.

Previous studies have shown that aminoglycoside antibiotics induce hair cell death through apoptosis (Forge and Schacht, 2000; Cunningham et al., 2002, 2004). To confirm that gentamicin-treated hair cells died via the apoptotic pathway in our organotypic cultures, whole-mount preparations were stained with TUNEL to assess apoptotic cell death. Accordingly, massive TUNEL signals were detected in MYO6+ hair cells in gentamicin-treated explants (Figure 1E). Additionally, in sections of gentamicin-treated organs of Corti, MYO6+ hair cell loss was obvious whereas supporting cells (labeled by anti-p27*^Kip^*^1^) remained intact (Figure 1F), indicating that gentamicin is primarily toxic to hair cells.

Together, our results obtained from gentamicin-treated organotypic cultures recapitulate the characteristics of aminoglycoside-induced hair cell death reported by others, including hair cell-specific uptake of gentamicin, basal-to-apical gradient of hair cell sensitivity, cell death through apoptosis, and the relative resistance of supporting cells (Forge and Schacht, 2000; Sha et al., 2001; Oesterle et al., 2008; Alharazneh et al., 2011). Since hair cells in the basal segment are more sensitive to gentamicin ototoxicity than hair cells in the apical region, we focused on the hair cell damage in the base for the remainder of this study; thus, all images henceforth of surface prep of cochleae were taken from the base regions unless otherwise noted.

### Inhibition of CDK2 activity confers a protective effect against gentamicin ototoxicity

**FIGURE 2:**
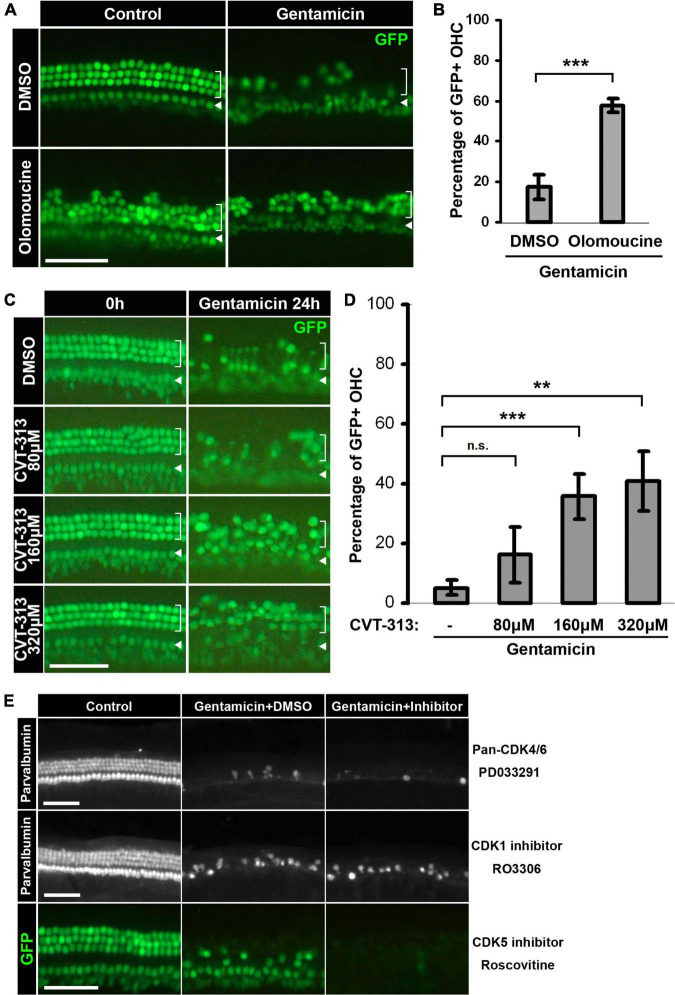
Inhibition of CDK2 activity confers protective effects against gentamicin ototoxicity. **(A)** Pan-CDK1,2,5 inhibitor Olomoucine protects hair cells from gentamicin-induced loss. Representative images of the base of cochleae are shown. Gentamicin treatment induces massive hair cell loss at 24-h, but more GFP-positive hair cells survive when Olomoucine is present. **(B)** Quantification of the percentages of GFP-positive outer hair cells in the base of the organs treated by gentamicin alone or by gentamicin and Olomoucine. ***p = 0.0018, *n* = 3. **(C)** CDK2 specific inhibitor CVT-313 protects hair cells from gentamicin in a dose-dependent manner. Representative images of the base of cochleae at the beginning of treatment and 24-h are shown. The protective effect of CVT-313 correlates with the concentration of CVT-313. **(D)** Quantification of the percentages of GFP-positive outer hair cells in the base of the organs at 24-h. For 80 μM CVT-313, adjusted-*p* = 0.34; for 160 μM, adjusted-*p* = 0.0074; for 320 μM, *p* = 0.012; *n* = 3. **(E)** No protective effect observed for Pan-CDK4/6 inhibitor PD0332991, CDK1 inhibitor RO3306 or CDK5 inhibitor Roscovitine. Hair cells are either labeled by Parvalbumin staining or marked by GFP. Inner hair cells are indicated by arrowheads, and outer hair cells are indicated by brackets. Scale bars, 50 μm. All surface prep pictures were taken from the base of the cochleae. Error bars in quantification plots represent standard deviations.

Cyclin-dependent kinase activity has been implicated in the cell death of postmitotic neurons, presumably either by promoting aberrant cell cycle re-entry (Folch et al., 2012) or by directly modulating apoptotic pathways (Konishi et al., 2002; Yuan et al., 2008; Vanden Bush and Bishop, 2011). We asked whether aminoglycoside ototoxicity is regulated by CDK activity by examining the effect of pharmaceutical CDK inhibitors in gentamicin-treated hair cells. We tested CDK1/2/5 pan-inhibitor Olomoucine (Veselı et al., 1994) and CDK4/6 pan-inhibitor PD0332991 (Fry et al., 2004) and charted hair cell numbers in the basal cochlear turn at 24 h post-treatment. Olomoucine appeared to confer a protective effect against gentamicin-induced hair cell loss, as evidenced by the greater number of OHCs in the presence of Olomoucine relative to DMSO control (Figures 2A,B). Quantification of this protective effect against gentamicin-induced OHC loss is shown in Figure 2B (57.8 ± 3.6% of OHCs in Olomoucine + Gent, 17.5 ± 6.0% in DMSO + Gent; *n* = 3; *p* = 0.0018). In contrast, PD0332991 failed to exert any protective effect even at the highest concentration tested (20 μM, which is >1,000 fold of IC50 for CDK4/6) (Figure 2E, top panel). The protective effect of Olomoucine suggests that CDK1, CDK2, and/or CDK5 may be involved in gentamicin-induced hair cell death.

To more specifically ascertain the CDK(s) involved in gentamicin-induced hair cell loss, we tested several other CDK inhibitors with high specificities against CDK1, CDK2, and CDK5 (Table 1). Of these, the CDK2-specific inhibitor CVT-313 (Brooks et al., 1997) appeared to protect hair cells against gentamicin-induced hair cell loss, as evidenced by an increase in OHC survival in the presence of CVT-313 compared to DMSO control (Figures 2C,D). The protective effect conferred by CVT-313 was dose-dependent (5.2 ± 2.5% viable OHCs in DMSO + Gent; 16.2 ± 9.2% in 80 μM CVT-313 + Gent, adjusted-*p* = 0.34; 35.8 ± 7.4% in 160 μM CVT-313 + Gent, adjusted-*p* = 0.074; 40.9 ± 10.0% in 320 μM CVT-313 + Gent, adjusted-*p* = 0.012; *n* = 3; Figure 2D). In contrast, neither RO3306, a selective inhibitor of CDK1 (Vassilev et al., 2006) nor Roscovitine, an inhibitor more potent for CDK5 (Meijer et al., 1997), conferred any protective effect against gentamicin-induced hair cell loss (Figure 2E, middle and bottom panels).

Additionally, both Olomoucine and CVT-313 protected hair cells from damage induced by 1 mM neomycin, another widely used aminoglycoside antibiotic (Supplementary Figure 2). Together, our data show that pharmaceutical inhibition of CDK2 confers protection against aminoglycoside ototoxicity, implicating a role for CDK2 activity in modulating aminoglycoside-induced hair cell death.

### Inhibition of CDK2 activity delays intrinsic apoptosis and suppresses c-Jun activity

**FIGURE 3:**
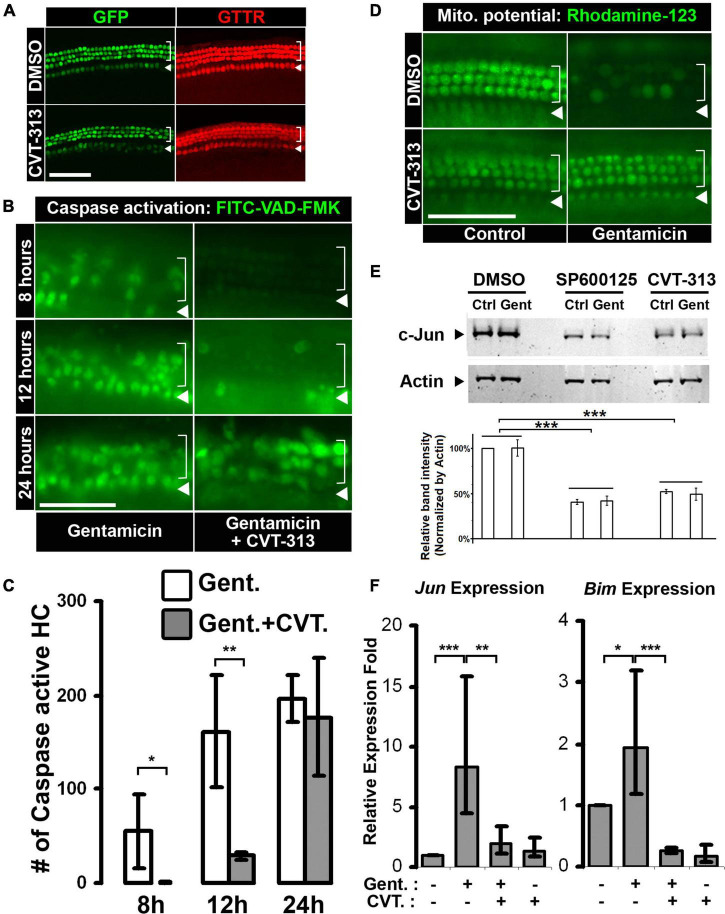
Inhibition of CDK2 activity delays gentamicin-induced hair cell apoptosis through c-Jun activity suppression. **(A)** Hair cell specific uptake of gentamicin is not affected by CDK2 inhibition. GTTR accumulates specifically in hair cells in the absence or presence of CDK2 inhibitor CVT-313. **(B)** Inhibition of CDK2 by CVT-313 delays gentamicin-induced activation of caspase cascade. Representative images of the base of the cochleae (wild-type, no *Atoh1-GFP* transgene) at 8-, 12-, and 24-h are shown. Activation of Caspase is indicated by FITC fluorescence, and there are more FITC-positive hair cells in organs treated by gentamicin alone than organs treated by gentamicin and CVT-313 at 8- and 12-h. **(C)** Quantification of FITC-positive hair cells in the base of cochleae at 8-, 12-, and 24-h timepoints. *p* = 0.076 for 8-h; *p* = 0.018 for 12-h; *p* = 0.64 for 24-h; *n* = 3. **(D)** Inhibition of CDK2 activity by CVT-313 preserves mitochondrial membrane potential in hair cells after gentamicin treatment. Representative pictures of the base of cochleae (wild-type, no *Atoh1-GFP* transgene) at 6-h are shown. Fluorescence of Rhodamine-123 indicates the existence of potential difference across the mitochondrial membrane. Gentamicin alone induces the loss of mitochondrial membrane potential in hair cells, while the presence of CVT-313 preserves the potential at 6-h. **(E)** Inhibition of CDK2 by CVT-313 reduces c-Jun protein in the whole organ of cochlea, shown by western blotting. No differences were detected between untreated (Ctrl) and gentamicin-treated (Gent) organs; but JNK inhibitor SP600125 and CDK2 inhibitor CVT-313 reduce c-Jun level significantly. Actin is used as loading control. The band intensities were measured by LI-COR Image Studio and normalized with Actin bands. ***Adjusted-*p* < 0.001, *n* = 3. **(F)** Quantitative-PCR showing gentamicin-induced *Jun* and *Bim* expression in hair cells are suppressed by CDK2 inhibitor CVT-313. *p < 0.1; **p < 0.05; ***p < 0.01. For images of surface preps, inner hair cells are indicated by arrowhead and outer hair cells are indicated by bracket. All surface prep images were taken from the base of the cochleae. Scale bar, 50 μm. Error bars in quantification plots indicate standard deviations.

Using pharmaceutical inhibitors, we found that inhibition of CDK2 activity protected hair cells against aminoglycoside-induced hair cell death. To dissect the molecular mechanisms underlying this protective effect, we first measured the uptake of Texas-Red labeled gentamicin in hair cells in both the presence and absence of CVT-313 but found no difference in GTTR accumulation (Figure 3A), indicating that inhibiting CDK2 activity does not influence the entry of gentamicin into hair cells.

We next investigated the progress of apoptosis modulated by CDK2 activity during aminoglycoside-induced hair cell death. Because the intrinsic mitochondria-caspase pathway has been implicated in aminoglycoside-induced hair cell death (Cunningham et al., 2002, 2004), we tested the effect of CDK2 inhibition on the events of this apoptotic process. The intrinsic pathway of apoptosis involves mitochondrial dysfunction and collapse, releasing factors that initiate a cascade of caspase activation (Jiang and Wang, 2004). We began by assessing caspase cascade activation utilizing an active caspase *in situ* marker, FITC-VAD-FMK, which becomes fluorescent after binding to active caspase (Jayaraman, 2003). We monitored FITC fluorescence in live gentamicin-treated cultures in the presence and absence of CDK2 inhibitor CVT-313. In the gentamicin alone group, we first observed FITC-positive hair cells around 8 h post-treatment, with increasing accumulation of FITC fluorescence at the 12- and 24-h timepoints (Figure 3B, Gent alone). However, in the presence of CVT-313, the initial wave of FITC fluorescence was not seen until the 12-h mark (Figure 3B, Gent + CVT-313), suggesting that activation of an apoptotic caspase cascade was delayed when CDK2 was inhibited. This delay of caspase activation was quantified by counting the FITC-positive hair cells in the basal segment, which showed statistically significant reduction in FITC-positive hair cells in the presence of CVT-313 at 8 h (*p* = 0.076) and 12 h (*p* = 0.018), relative to the gentamicin alone control (Figure 3C; 55.0 ± 39.9, 161.3 ± 59.0, and 196.3 ± 24.6 FITC-positive hair cells in the Gent + DMSO group at 8, 12, and 24 h, respectively; 0.3 ± 0.6, 28.6 ± 3.2, and 177.0 ± 62.2 FITC-positive hair cells in the Gent + CVT-313 group at 8, 12, and 24 h, respectively).

It is known that collapse of mitochondrial integrity precedes the activation of caspase cascade during apoptosis (Jiang and Wang, 2004), thus we sought to determine the effect of CDK2 inhibition on gentamicin-induced collapse of mitochondria. The negative mitochondrial membrane potential is a universal indicator of mitochondrial integrity; thus, we used a fluorescent cationic dye, Rhodamine-123, which accumulates inside active mitochondria due to the existence of negative membrane potential (Baracca et al., 2003), to assess mitochondrial dysfunction and collapse. In control organs, nearly every hair cell showed high Rhodamine-123 fluorescence intensity at 6 h (Figure 3D, DMSO control; inner hair cells were out of focus). In contrast, Rhodamine-123 fluorescence diminished in gentamicin-treated hair cells (Figure 3D, DMSO gentamicin), indicating the collapse of mitochondria and the loss of membrane potential after gentamicin treatment. However, inhibiting CDK2 activity preserved the mitochondrial membrane potential, as evidenced by high Rhodamine-123 fluorescence in hair cells in the presence of CVT-313 at 6 h (Figure 3D, CVT-313 gentamicin panel). This observation indicates that CDK2 activity regulates signaling pathways upstream of mitochondrial collapse.

Since the JNK pathway has been implicated in hair cell apoptosis after ototoxin treatment (Pirvola et al., 2000; Ylikoski et al., 2002), and furthermore is a well-known regulatory mechanism of the expression of proapoptotic genes that initiate mitochondrial collapse (Dhanasekaran and Reddy, 2008), we investigated whether CDK2 activity regulates hair cell apoptosis through the JNK pathway. To assay the effect of CDK inhibition on the JNK pathway, we first measured the protein level of c-Jun, a phosphorylation substrate of JNK and a transcription factor of proapoptotic genes (Leppä and Bohmann, 1999), using western blotting. We found no significant difference between control and gentamicin-treated organs in each inhibitor group, and surmised that the limited number of hair cells (<2% of the cells in the cochlea) was the reason for our failure to detect the changes induced by gentamicin at the protein level using whole organ lysate. However, compared to DMSO control, inhibition of CDK2 by CVT-313 significantly reduced the protein level of c-Jun in the cochlea, similarly to JNK inhibitor SP600125 (Bennett et al., 2001), regardless of the presence of gentamicin (Figure 3E), indicating that inhibition of CDK2 activity reduces c-Jun at the protein level.

To further characterize the effect of CDK2 inhibition on the JNK pathway in hair cells, we purified hair cells after gentamicin and CVT-313 treatment and analyzed the transcription levels of two c-Jun target genes, *Jun* itself and *Bim*, by quantitative-PCR. Our results show that gentamicin alone induced the expression of both *Jun* and *Bim* (Figure 3F); however, this expression was suppressed by CDK2 inhibitor CVT-313, indicating that blocking CDK2 activity suppresses the gentamicin-mediated transcriptional activation of c-Jun target genes. We did not find statistically significant expression changes for *Bax* and *Gapdh* upon treatment with CVT-313 (Supplementary Figure 3), excluding the possibility that transcription suppression was due to the inhibition of general transcription apparatus through CDK7/9 activity blockage (Fu et al., 1999; Fisher, 2005).

Taken together, our data show that inhibiting CDK2 activity leads to the reduction of c-Jun at the protein level and suppresses the transcription of c-Jun target genes, which in turn delays the loss of mitochondrial membrane potential and caspase cascade activation induced by aminoglycoside ototoxicity.

### *Cdk2* inactivation by gene knockout desensitizes cochlear hair cells to aminoglycoside ototoxicity

One caveat in our pharmaceutical inhibitor studies is the potential off-target effects of the drugs. We therefore further investigated the role of CDK2 in aminoglycoside-induced hair cell death and hearing loss by knocking out the *Cdk2* gene under both *ex vivo* and *in vivo* conditions.

**FIGURE 4:**
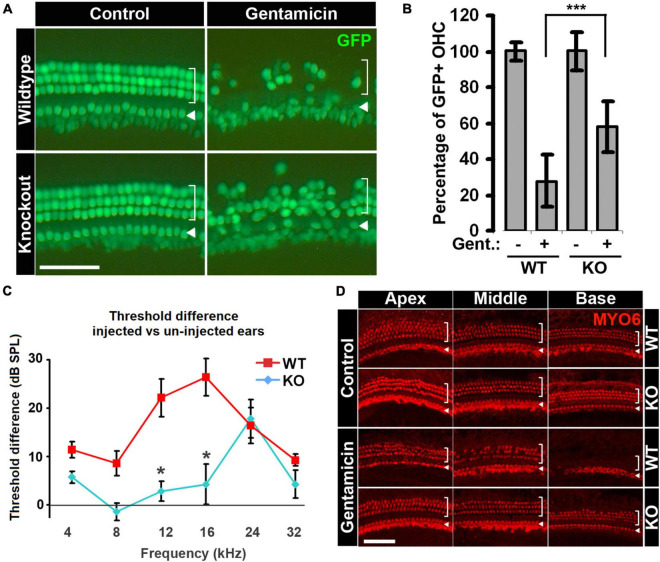
Hair cells in *Cdk2*–/– animals are less sensitive to gentamicin ototoxicity. **(A)** More hair cells survive gentamicin treatment in organotypic cultures of the organ of Corti from *Cdk2*–/– animals. Representative images of the base of cochlea with or without gentamicin treatment are shown. Hair cells are not affected by *Cdk2* deficiency in untreated control organs, and more hair cells survive gentamicin treatment in *Cdk2*–/– organs. **(B)** Quantification of percentages of outer hair cells in *Cdk2*+/+ and *Cdk2*–/– organs with or without gentamicin treatment. For the comparison of gentamicin-treated organs between genotypes, *p* = 0.0008, *n* = 8. ***p < 0.01. **(C)** Gentamicin injection-induced ABR threshold shifts are smaller in *Cdk2*–/– animals than *Cdk2*+/+ littermates. The differences between two genotypes at 12 and 16 kHz are statistically significant with *p* = 0.0549 and *p* = 0.0538, respectively; *n* = 7. *p < 0.1. **(D)** Gentamicin injection induces milder hair cell loss in *Cdk2*–/– animals compared to wild-type littermates, shown by immunostaining for MYO6. No difference is observed in untreated ears between genotypes (control panel), but hair cell loss is severe in *Cdk2*+/+ animals in gentamicin-injected ears compared to *Cdk2*–/– animals (gentamicin panel). Representative pictures of the apex, middle and base of cochleae are shown. For images of surface preps, inner hair cells are marked by arrowhead and outer hair cells are marked by bracket. Scale bars, 50 μm. Error bars in panels **(B,C)** indicate standard deviations.

For our *ex vivo* studies, explant cultures of neonatal cochleae from *Cdk2*−/− and *Cdk2*+/+ littermates were treated with gentamicin, and outer hair cells were quantified to compare their sensitivities to ototoxic insult. Untreated *Cdk2*−/− organs displayed a normal complement of outer and inner hair cells through the entire cochlear turn (Figure 4A), and their numbers were indistinguishable from those of wild-type explants (Figure 4B; 38.9 ± 4.1 OHC/100 μm in *Cdk2*−/− and 38.8 ± 1.8 OHC/100 μm in *Cdk2*+/+; *n* = 8). However, after gentamicin treatment, the basal turn of *Cdk2*−/− explants presented significantly more outer hair cells (58.0 ± 14.2%) relative to *Cdk2*+/+ explants (27.6 ± 14.4%, *n* = 8, *p* < 0.01; Figures 4A,B). The lower vulnerability of *Cdk2*-/- explants to gentamicin-induced hair cell loss indicates that knockout of the *Cdk2* gene confers a protective effect against gentamicin ototoxicity *ex vivo*.

To test whether *Cdk2* knockout also protects hearing *in vivo* following gentamicin treatment, we administered a single-dose of gentamicin by trans-tympanic injection (Xu et al., 2010) to the right ears of three-week-old animals, with the left ears serving as untreated controls. Two weeks after injection, we measured the hearing threshold differences between injected and un-injected ears using auditory brainstem response (ABR). There were no significant differences in hearing thresholds between the two genotypes in un-injected left ears (Supplementary Figure 4). For the injected right ears, *Cdk2*−/− mice manifested modestly elevated ABR thresholds at 24 kHz compared to un-injected left ears (*p* < 0.01; Figure 4C). In contrast, *Cdk2*+/+ animals showed elevated hearing thresholds at all frequencies tested in the injected right ears compared to un-injected left ears (Figure 4C; 5.7 ± 1.2 dB in KO vs 11.4 ± 1.7 dB in WT at 4 kHz; −1.4 ± 1.8 dB in KO vs 8.6 ± 2.5 dB in WT at 8 kHz; 2.8 ± 2.1 dB in KO vs 22.1 ± 3.9 dB in WT at 12 kHz; 4.2 ± 4.1 dB in KO vs 26.4 ± 3.8 dB in WT at 16 kHz; 17.9 ± 4.0 dB in KO vs 16.4 ± 3.7 dB in WT at 24 kHz; 4.3 ± 2.9 dB in KO vs 9.3 ± 1.3 dB in WT at 32 kHz; *n* = 7). The smaller threshold elevations observed in *Cdk2*−/− mice indicate that, compared to wild-type littermates, the hearing loss of *Cdk2*−/− mice was alleviated from gentamicin treatment.

To confirm that the alleviated hearing loss in the *Cdk2*−/− mice was the result of reduced hair cell death, we examined cochlear hair cells in *Cdk2*−/− and *Cdk2*+/+ mice after gentamicin injection by MYO6 immunostaining. Compared to wild-type controls, there were no abnormalities throughout the entire cochlear turn in the un-injected contralateral ears of *Cdk2*−/− animals (Figure 4D, Control panel). Injected right ears of *Cdk2*+/+ animals manifested severe hair cell loss in a basal-to-apical gradient (Figure 4D, Gentamicin panel, WT); however, hair cell loss in the injected ear of *Cdk2*−/− animals was much milder, with a few hair cells missing in the base (Figure 4D, Gentamicin panel, KO). This piece of evidence confirms the role of CDK2 activity in modulating the sensitivity to gentamicin-induced hearing loss via regulating cochlear hair cell damage *in vivo*.

Taken together, our data indicate that removal of CDK2 activity by knocking out the *Cdk2* gene desensitizes cochlear hair cells to aminoglycoside ototoxicity both *ex vivo* and *in vivo*, strongly supporting our hypothesis that CDK2 activity regulates aminoglycoside-induced hair cell death.

## Discussion

Due to the lack of regenerative capacity in the mammalian cochlear sensory epithelium, hearing loss caused by ototoxin-induced hair cell damage and death is permanent in humans (Kwan et al., 2009; Groves, 2010). Thus, tremendous efforts have been dedicated to understanding the molecular mechanisms of hair cell death induced by ototoxic side effects of drugs that are widely used in clinical practice, including aminoglycoside antibiotics. Oxidative stress is thought to be the major trigger of the apoptosis pathway during aminoglycoside-induced hair cell death, with mitochondrial dysfunction considered the main source of damaging oxidative species (Hirose et al., 1997; Sha and Schacht, 1999; Jensen-Smith et al., 2012; Schacht et al., 2012). However, our previous transcriptome analysis revealed several thousand genes significantly up- or downregulated in gentamicin-treated hair cells prior to any transcriptional response to oxidative stress (Tao and Segil, 2015). Thus, we inferred that the progression of aminoglycoside-induced hair cell death relies on additional signaling pathways upstream of mitochondrial collapse. Based on the observations that cell cycle-related genes are significantly upregulated by gentamicin treatment in hair cells (Tao and Segil, 2015) and given the fact that suppression of cell cycle machinery is critical for hair cell survival (Chen et al., 2003; Laine et al., 2007), we speculated that key cell cycle proteins such as CDKs contribute to the death of postmitotic hair cells after ototoxic insult. Indeed, multiple groups have independently discovered the protective effects of CDK inhibitors against ototoxin-induced hair cell loss. Ryals et al. (2017) found that CDK4 inhibitors protected hair cells against gentamicin in organ explants. Teitz et al. (2018) identified CDK2 inhibitors as protectants against cisplatin- and noise-induced hair cell death. In addition, unpublished data from our colleague Menendez revealed the protective effect of CDK inhibitors against cisplatin and gentamicin in reprogrammed hair cell-like cells (unpublished data). However, there remain some inconsistencies among the findings of these groups. Ryals et al. found CDK1/2 inhibitors non-protective yet CDK4 inhibitors protective, contradicting the findings from both Teitz et al. and our group. We suspect this discrepancy may be due to differences in experimental procedures. Ryals et al. (2017) preincubated explants with inhibitors for 24 h, which potentially changed the expression of many genes. Teitz et al. (2018) only pretreated organs with inhibitors for 1 h and we did not preincubate explants with inhibitors. The specificity and concentrations of inhibitors might also account for inconsistencies. Despite such discrepancies, all these observations taken together support the hypothesis that cell cycle machinery, especially CDK activity, regulates hair cell death after ototoxic stimuli.

We took further steps to investigate the underlying signaling pathways targeted by CDK2 during ototoxin-induced hair cell death. Initially, we speculated that CDK activity promoted hair cell death through aberrant cell cycle re-entry and checkpoint activation based on previous findings that the knocking out of CKI genes leads to aberrant cell cycle re-entry and eventually apoptosis in hair cells (Chen et al., 2003; Laine et al., 2007). However, we failed to detect cell cycle re-entry in hair cells by EdU incorporation after gentamicin treatment (Supplementary Figure 5). Furthermore, our immunostaining assays did not yield a positive signal for CHK2 phosphorylation, which is a marker of checkpoint activation (Bartek and Lukas, 2003), in hair cells after gentamicin treatment (data not shown). In addition, knocking out of *p53* gene, a critical mediator of cell cycle checkpoint activation-triggered apoptosis (Fridman and Lowe, 2003; Giono and Manfredi, 2006), did not affect gentamicin-induced hair cell death (Supplementary Figure 5). Based on this data, we concluded that CDK2 activity does not regulate hair cell death by encouraging cell cycle re-entry.

Alternatively, various lines of evidence support the possibility that CDK2 regulates aminoglycoside-induced hair cell death in a manner independent of the cell cycle. Multiple non-cell cycle proteins have been identified as direct phosphorylation substrates of CDKs during apoptosis of postmitotic cells, including BAD, FOXO1, and c-Jun. Proapoptotic factor BAD has been found to be phosphorylated by CDK1, after which it is released from 14-3-3 sequestration, leading to apoptosis (Konishi et al., 2002). FOXO1 protein has been identified as another substrate of CDK1. Phosphorylated FOXO1 accumulates in nuclei, stimulating expression of proapoptotic genes and initiating cell death in neurons (Yuan et al., 2008). Phosphorylation of the JNK pathway substrate c-Jun by CDKs has also been discovered in differentiated postmitotic immune cells (Ghahremani et al., 2002; Vanden Bush and Bishop, 2011). Here, we examined the relationship between CDK2 and c-Jun in ototoxin-induced hair cell death and found that inhibition of CDK2 decreased c-Jun at the protein level and suppressed the transcription of c-Jun target genes. It has been demonstrated that phosphorylation of c-Jun reduces ubiquitination-dependent c-Jun degradation (Musti et al., 1997) and that phosphorylation of the c-Jun N-terminus enhances its transcriptional activity (Binétruy et al., 1991; Pulverer et al., 1991). We failed to detect the decrease in phosphorylation of c-Jun at S73 after CDK2 inhibition (Supplementary Figure 3); however, it is possible that CDK2 directly phosphorylates c-Jun at other sites in hair cells after aminoglycoside treatment.

The expression of proapoptotic BH3-only protein BIM is critical during neuronal cell death (Putcha et al., 2001; Whitfield et al., 2001). It has been found that BIM expression is co-regulated by Myb, FoxO, and c-Jun, all three of which are subject to modulation by CDK activity (Biswas et al., 2007; Greene et al., 2007). We found that expression of *Bim* was induced in hair cells after gentamicin treatment and that this expression induction was suppressed by CDK2 inhibition. We think it is possible that in addition to c-Jun, CDK2 also modulates other pathways to indirectly regulate *Bim* expression.

We tested multiple cyclin-dependent kinase by either pharmaceutical inhibition or gene knockout, finding CDK2 to be significantly involved in aminoglycoside-induced apoptosis of hair cells. Inhibition of CDK5 by Roscovitine did not protect hair cells against gentamicin. Contrarily, it has been found that removal of CDK5 activity by conditional knockout of the *Cdk5* gene in hair cells leads to hair cell death (Zhai et al., 2018), suggesting that CDK5 activity is required for hair cell survival. The function of CDK5 during ototoxin-induced hair cell death remains an open question. CDK1 inhibitor RO3306 also did not show any protective effect in our assay, even though CDK1 and CDK2 have been found to be redundant (Aleem et al., 2005; L’Italien et al., 2006) and share many of the same substrates (Chi et al., 2008; Errico et al., 2010), and furthermore CDK1 has been found to regulate neuronal cell death (Konishi et al., 2002; Yuan et al., 2008). It is still possible that CDK1 participates in the hair cell apoptosis process after ototoxic stimuli in a yet unidentified manner. In addition to testing CDK4/6 inhibitor PD0332991, which displayed no protective effect, we also compared the effect of gentamicin on *Cdk4*−/− and *Cdk4*+/+ animals and did not find any significant difference in numbers of GFP-positive OHCs 24 h post gentamicin treatment (Supplementary Figure 5), suggesting that CDK4 activity was not involved in aminoglycoside-induced hair cell death.

One caveat of this study using organotypic cultures of cochleae from neonatal animals is that the organ of Corti is still under development at this stage and the hair cells are not yet considered fully functional. As stated, we focused our study on the basal segment of the cochlea, as the gentamicin-induced damage observed in the apex was mild (Supplementary Figure 1). The absence of GTTR (Figure 1A) and the resistance to gentamicin ototoxicity in apical hair cells (Figure 1B and Supplementary Figure 1) might be due to the underdeveloped transduction channels which have been shown to be the route of gentamicin entry into hair cells (Alharazneh et al., 2011). Our *in vivo* study of *Cdk2* knockout mice overcomes this as well other possible age-related caveats, as we used three-week-old mice, whose auditory organs are defined as mature and fully functional. We showed that knocking out the *Cdk2* gene provides protection against gentamicin ototoxicity in these animals, corroborating our discoveries with neonatal *Cdk2*−/− cultures. In brief, removal of CDK2 activity by both pharmaceutical inhibition at the protein level and by *Cdk2* gene knockout provides protection against aminoglycoside-induced hair cell death. Importantly, however, neither of these methods completely rescued cells from toxin-induced death, so it is possible there are other routes by which aminoglycoside ototoxicity induces hair cell death, such as disruption of intracellular calcium homeostasis (Esterberg et al., 2013) or elevation of intracellular reactive oxygen species (Schacht, 1999).

In this study, we identify a regulatory role of CDK2 in aminoglycoside antibiotic-induced sensory hair cell death, revealing a promising target for preventing clinical drug-induced hearing loss. The potential therapeutic application of CDK inhibitors in preventing hearing loss is further supported by the finding that inhibition of CDK activity protects spiral ganglion cells, which innervate cochlear hair cells and project to cochlear nuclei, against apoptosis secondary to sensory hair cell death (Liu et al., 2016). The therapeutical efficacy of CDK inhibitor in preventing aminoglycoside-induced hearing loss requires further investigation.

## Data availability statement

The original contributions presented in this study are included in the article/Supplementary material, further inquiries can be directed to the corresponding author.

## Ethics statement

The animal study was reviewed and approved by IACUC, House Research Institute; IACUC, University of Southern California.

## Author contributions

LT performed the experiments, wrote, and edited the manuscript. NS acquired the fund for this project. Both authors conceived this project, analyzed the data, designed the experiments, contributed to the article, and approved the submitted version.
