# Supplementary material for: CDK2 regulates aminoglycoside-induced hair cell death through modulating c-Jun activity: Inhibiting CDK2 to preserve hearing
Source: Front Mol Neurosci. 2022 Oct 13;15:1013383. doi: 10.3389/fnmol.2022.1013383 (PMC9606710; doi:10.3389/fnmol.2022.1013383)
Supplement: Supplementary file 1 [file Data_Sheet_1.PDF]

Supplementary Figures

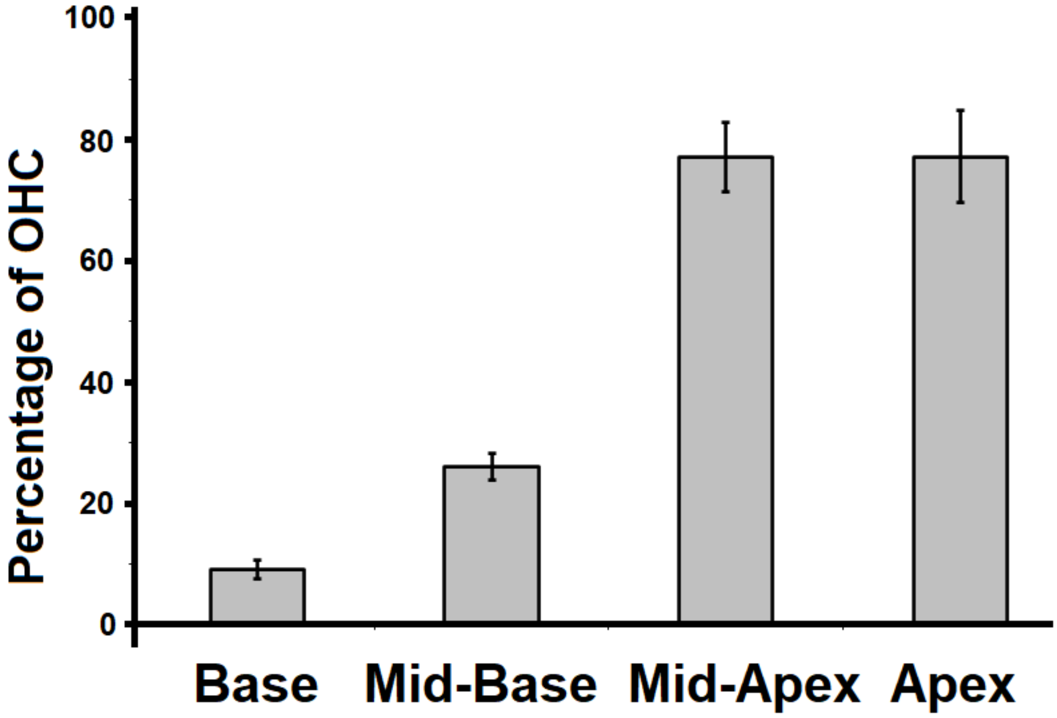

Figure S1. Gentamicin induces hair cell loss with a base-to-apex gradient. Quantification of remaining GFP-positive outer hair cells at different segments of the cochlea 24 hours after gentamicin treatment. Error bars stand for standard deviation. n = 3.

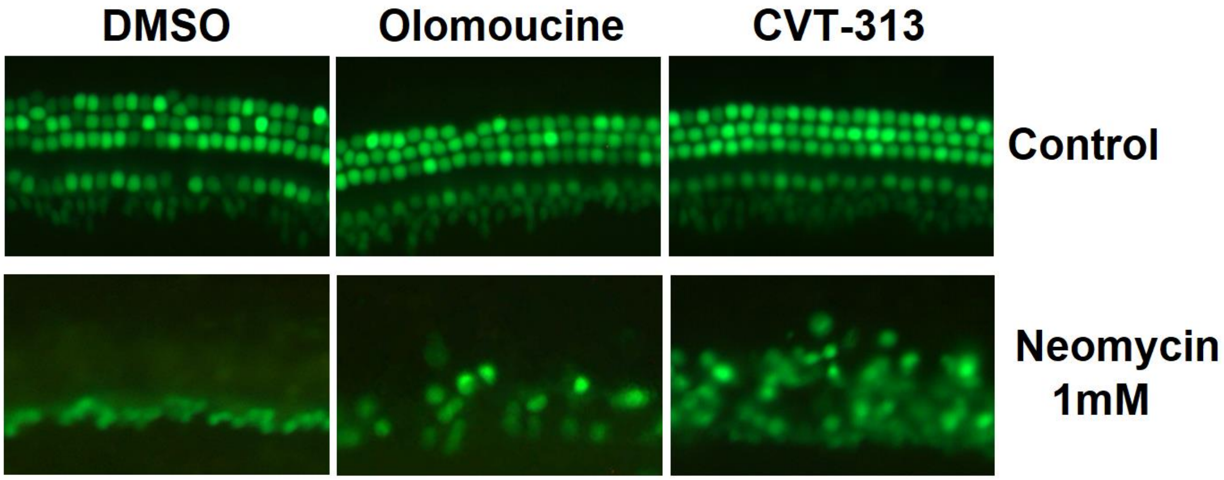

Figure S2. CDK2 inhibitor Olomoucine and CVT -313 protect hair cells against neomycin. Representative picture of the basal regions of the cochleae 24 hours after 1mM neomycin treatment. Hair cells are labeled by GFP.

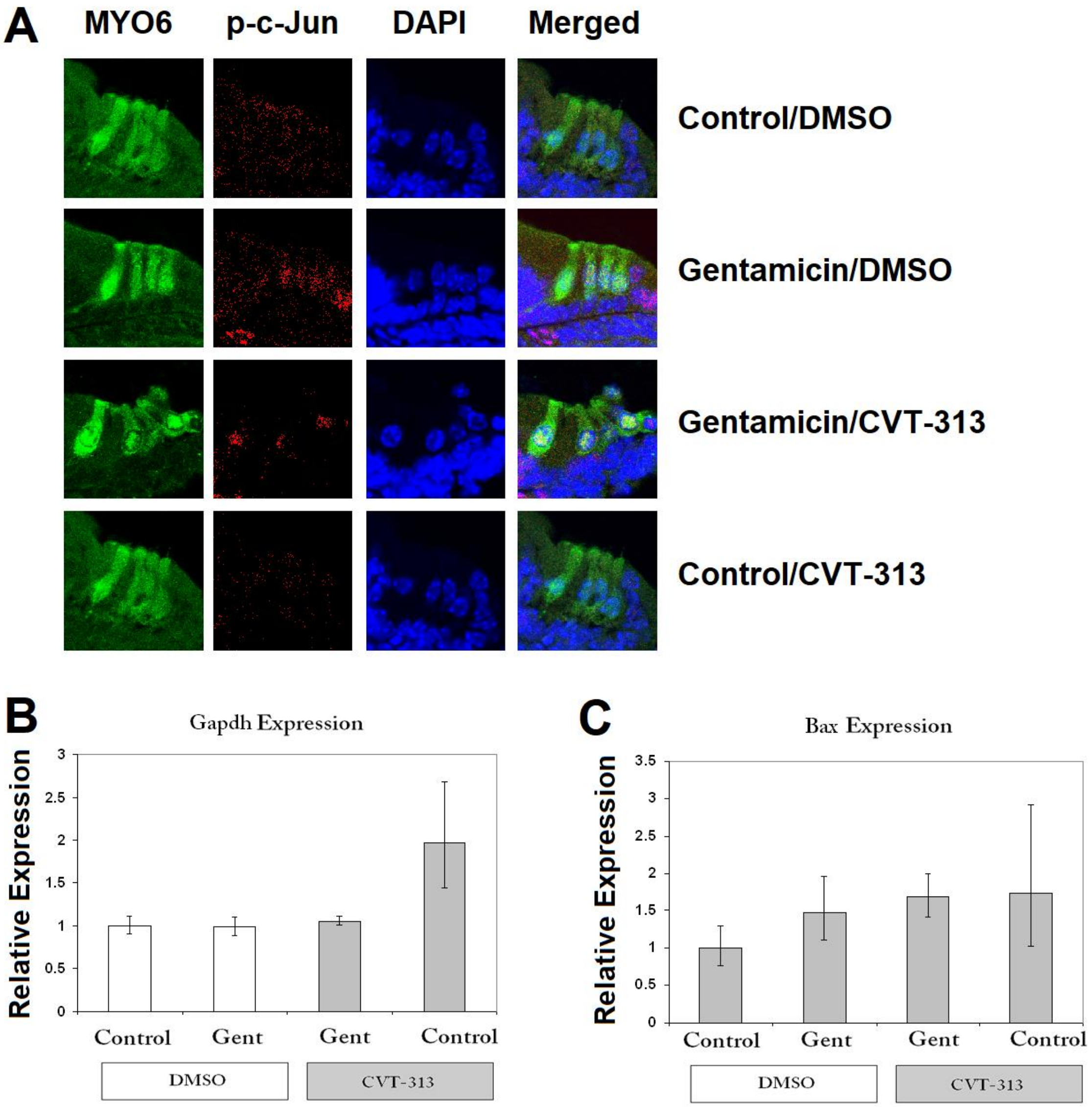

Figure S3. CDK2 inhibition does not affect gentamicin-induced c-Jun phosphorylation or expression of *Gapdh* and *Bax*. A, immunostaining of p-c-Jun in hair cells after gentamicin treatment with/without the presence of CDK2 inhibitor CVT-313. Hair cells are co-stained with MYO6. Gentamicin treatment induces phosphorylation of c-Jun at S73, as indicated by nuclear staining of p-c-Jun (red); however, S73 phosphorylation of c-Jun is not affected by CVT-313. B and C, the expressions of *Gapdh* and *Bax* genes in hair cells are not suppressed by CDK2 inhibitor CVT-313, suggesting that general transcription machinery is not inhibited by CVT-313. Expression of *Rpl19* was used as internal control for relative expression quantification by q-PCR.

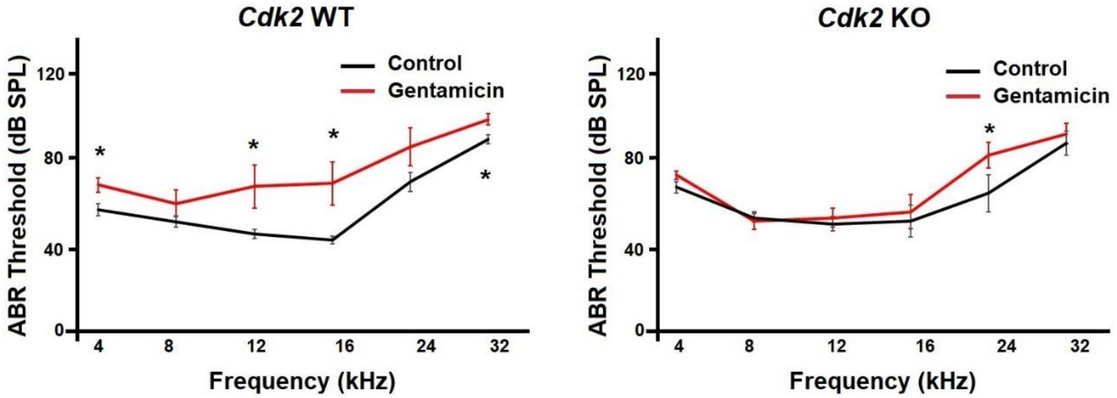

Figure S4. ABR threshold of un-injected (Control) and gentamicin-injected (Gentamicin) ears of *Cdk2* WT and KO animals. Error bar, standard error; *, $p < 0.05$ from student t-test; n = 7.

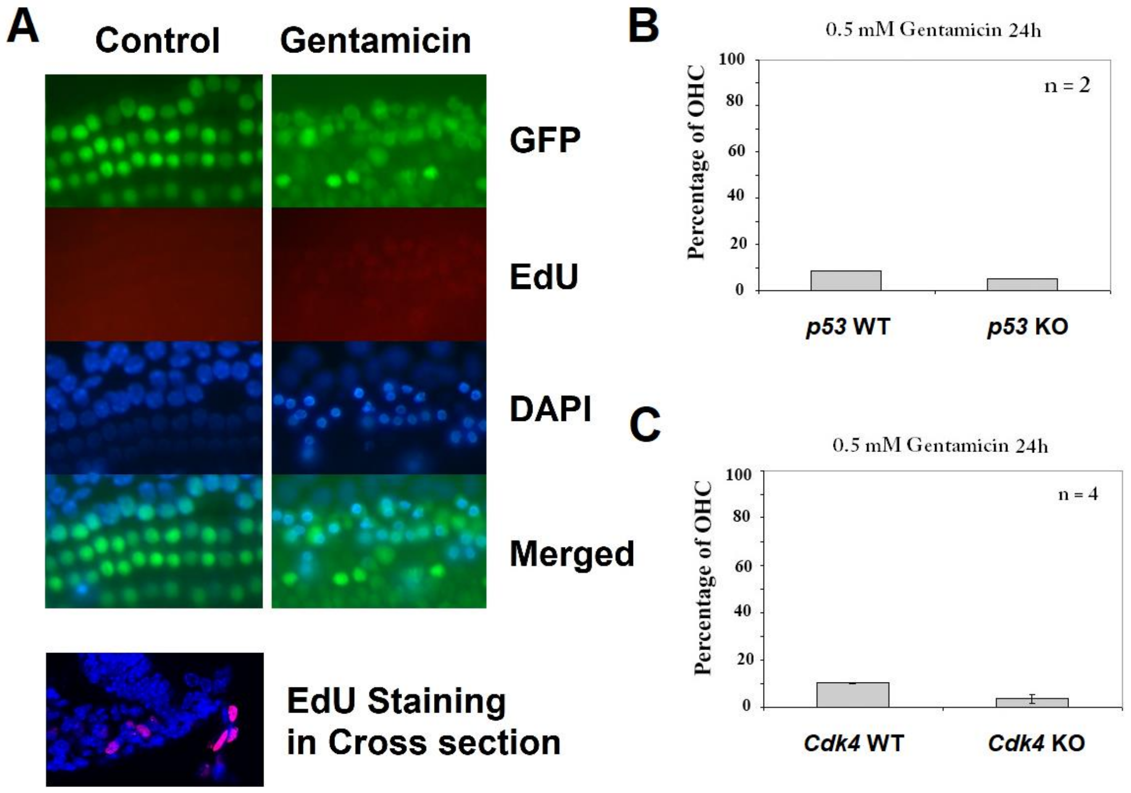

Figure S5. Aberrant cell cycle re-entry does not contribute to gentamicin-induced hair cell death. A, EdU incorporation was not detected in hair cells after gentamicin treatment, indicating the absence of DNA synthesis. Images were taken with over-exposure in red channel. A cross section with positive EdU signals in cells outside of the organ of Corti was shown as the positive control for EdU staining procedures. B, 24 hours after 0.5mM gentamicin treatment, similar numbers of outer hair cells were found in the base of the cochleae from *p53* WT and KO organs. C, there is no protection against gentamicin-induced hair cell death by knocking out of *Cdk4* gene, as indicated by similar percentages of remaining outer hair cells at the base of gentamicin-treated WT and KO organs 24 hours after treatment.
